# Cross-ecosystem engineers: the dual role of fruit bats in cave nutrient dynamics and landscape seed dispersal

**DOI:** 10.1007/s00442-026-05930-5

**Published:** 2026-07-20

**Authors:** Maricélio de Medeiros Guimarães, Priscila Emanuela de Souza, Marconi Souza-Silva, Rodrigo Lopes Ferreira

**Affiliations:** Center of Studies on Subterranean Biology, Department of Ecology and Conservation, Federal University of Lavras, Lavras, 37200-90 Minas Gerais Brasil

**Keywords:** Chiroptera, Subterranean ecosystem, Ecosystem services, Ecological functional, Neotropics

## Abstract

Bats provide key ecosystem services, yet their dual role in subterranean habitats remains understudied. Frugivorous species such as *Artibeus planirostris* can act both as seed dispersers in surface environments and as sources of organic matter in caves through guano deposition. We investigated these functions in Toca do Morrinho Cave (Bahia, Brazil), combining captive experiments and field monitoring to assess guano production, diet, deposition patterns, and seed fate. Bats consumed fruits from 11 plant species and produced an average of 3.24 g of dry guano per day. Most feces (91.1%) were deposited outside the cave, corresponding to ~ 275 seeds per bat per day, underscoring their efficiency as seed dispersers. In contrast, only 8.9% of feces were dropped inside the cave, where seeds failed to germinate under aphotic conditions. Nevertheless, this deposition enriched the hypogean environment, providing organic matter and moisture that support invertebrate assemblages. Germination trials showed that gut passage generally preserved or enhanced seed viability, particularly for *Ficus gomelleira*. Our results highlight the multifunctionality of *A. planirostris* as both a driver of plant recruitment across landscapes and a contributor to subterranean nutrient dynamics. While seed dispersal is highly effective in epigean systems, deposition underground represents an antagonistic outcome for plants but a critical subsidy for cave communities. By linking above- and belowground ecosystems, *A. planirostris* functions as a cross-ecosystem engineer. Protecting its cave colonies is therefore vital not only for subterranean biodiversity but also for broader ecological processes that depend on bat-mediated connectivity.

## Introduction

Ecosystem services provided by animals are fundamental to ecosystem functioning (Sekercioglu 2010). Among vertebrates, bats are particularly notable because they contribute to multiple categories of ecosystem services, including seed dispersal, pollination, insect population regulation, and the transfer of nutrients across ecosystem boundaries (Fenton et al. 1992; Kunz et al. 2011; Ghanem and Voigt 2012; Ferreira 2019; Nsengimana et al. 2023; Liu et al. 2025). Despite their ecological relevance, the importance of bats is often underestimated, largely due to limited awareness of the diversity of their feeding strategies and functional roles (Boyles et al. 2011; Kunz et al. 2011).

In addition to their well-known roles in plant reproduction and insect regulation, bats can also mediate energy transfer between ecosystems. In subterranean environments, where primary production is absent and energy inputs are typically limited, bats often represent a major source of allochthonous organic matter through the deposition of guano, urine, and carcasses (Poulson 1972; Ferreira and Martins 1998, 1999; Ferreira et al. 2000; Ferreira 2019). These inputs sustain diverse cave communities and form the basis of complex detrital food webs, particularly in permanently dry caves where alternative sources of organic matter are scarce (Ferreira and Martins 1998, 1999; Dainelli et al. 2025). Over long temporal scales, the accumulation and decomposition of guano may also modify cave environments, driving chemical corrosion processes that can significantly alter cave morphology (Piló et al. 2023).

Frugivorous bats play a central role in tropical ecosystems by dispersing seeds across landscapes and contributing to plant recruitment and forest regeneration (Morrison 1978; Passos and Passamani 2003; Oliveira and Lemes 2010; Kunz et al. 2011). Many species consume fruits during nocturnal foraging and later defecate seeds at considerable distances from parent plants, often enhancing seed dispersal effectiveness (Morrison 1978; Passos and Passamani 2003). However, when frugivorous bats use caves as roosts, part of the consumed plant material may be deposited within subterranean environments, where seeds may germinate but cannot develop due to the absence of light in aphotic zones (Fadini and Castro 2013). In such cases, plant-bat interactions may shift from mutualistic to antagonistic, since seed deposition does not result in successful plant establishment (Nogueira and Peracchi 2003; Jordano et al. 2006).

Caves are commonly used as roosts by numerous bat species worldwide (Kunz 1982). In Brazil, which harbors at least 186 bat species (Garbino et al. 2024), a proportion of 44% has been recorded using caves either as permanent shelters or temporary refuges (Barros and Bernard 2023), and at least 42 of these species are strictly frugivorous (Enrico Bernard, pers. comm. 2025). Among them, several frugivorous species may contribute simultaneously to seed dispersal in epigean environments and to nutrient inputs within subterranean ecosystems by depositing feces inside caves (Bredt et al. 1999; Esbérard et al. 2005; Sbragia and Cardoso 2008; Luo et al. 2013; Sakoui et al. 2020). This dual ecological role highlights the potential for bats to link surface and subterranean ecosystems through the movement of organic matter and propagules.

Among Neotropical frugivorous bats, *Artibeus planirostris* Spix, 1823 is a medium-sized species (40–69 g; forearm 62–73 mm) with a broad diet that includes fruits, leaves, floral resources, and insects (Zortéa 2007; Horsley et al. 2015; Cordero-Schmidt et al. 2016). Its rapid gut passage and frequent production of viable seeds make it an effective seed disperser (Morrison 1978; Passos and Passamani 2003; Oliveira and Lemes 2010; Silveira et al. 2024). The species has a wide distribution across South America and occurs in several Brazilian biomes, where it typically roosts in tree canopies but may also use caves (Gregorin and Mendes 1999; Coelho 2006; Barquez and Diaz 2015; Cordero-Schmidt et al. 2016; Bordignon and Shapiro 2017; Torquetti et al. 2017).

Within this framework, a potential trade-off emerges between two important ecosystem services provided by frugivorous bats: seed dispersal and nutrient input into subterranean systems. When feces are deposited outside caves, bats function primarily as effective seed dispersers. In contrast, defecation within caves contributes mainly to the enrichment of subterranean ecosystems through organic matter input. Based on this premise, the present study investigates the ecological role of *Artibeus planirostris* in caves, focusing on its dual function as both a seed disperser in epigean habitats and a contributor of organic matter to hypogean environments.

## Materials and methods

### Species and study area

In Brazil, *A. planirostris* has been recorded across several regions and biomes, reflecting its broad geographic distribution within the country (Coelho 2006; Cordero-Schmidt et al. 2016; Bordignon and Shapiro 2017). The species typically roosts in tree canopies; however, the use of caves as shelter has also been documented in different parts of its range (Gregorin and Mendes 1999; Coelho 2006; Sbragia and Cardoso 2008; Torquetti et al. 2017).

In karst landscapes of the Cerrado (Brazilian savanna), records of *A. planirostris* are relatively scarce, with only two reports from cave environments to date (Cunha et al. 2009; Torquetti et al. 2017). In contrast, the species has been documented in several caves within the Caatinga biome (Gregorin and Mendes 1999; Coelho 2006; Sbragia and Cardoso 2008; Falcão 2020). Some colonies also exhibit notable long-term persistence. For example, the population inhabiting Morrinho Cave, in northern Bahia, has reportedly occupied the site continuously for at least three decades (Rodrigo L. Ferreira, pers. comm. 2025). It is important to note that bat diversity and community composition vary substantially among Brazilian biomes. Despite its ecological uniqueness, the Caatinga remains comparatively underexplored from a speleological perspective, and knowledge of its cave-dwelling bat assemblages is still limited (Falcão 2020; Mendes and Srbek-Araujo 2020).

**Fig. 1:**
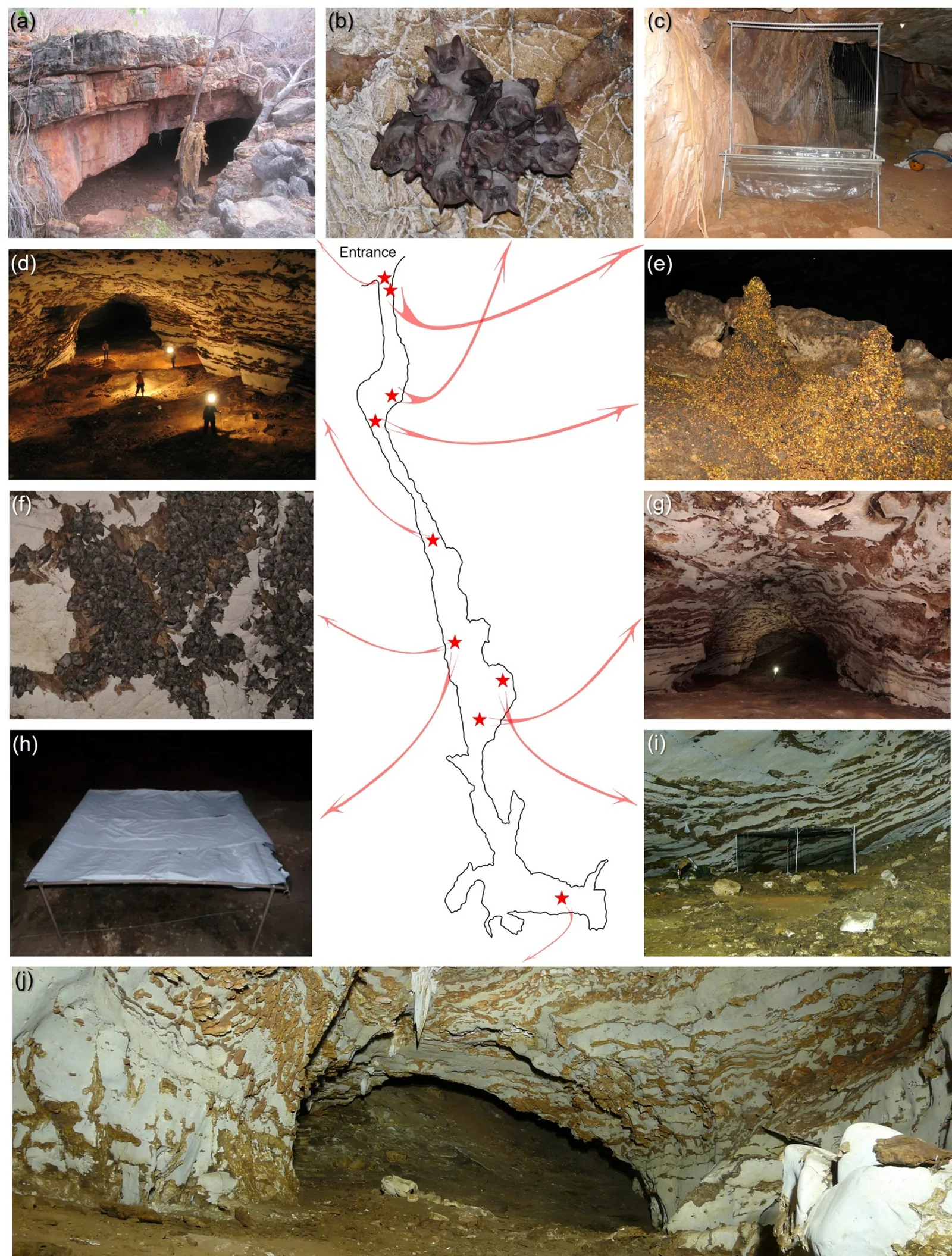
Ground plan of Toca do Morrinho Cave, Campo Formoso, Bahia, Brazil (modified from Ferreira et al. 2007), with illustrative images: (**a**) cave entrance; (**b**) *Artibeus planirostris* roosting in Chamber I; (**c**) harp traps; (**d**) Chamber III; (**e**) fruigivorous *A. planirostris* guano deposits forming small guanites.; (**f**) *A. planirostris* individuals; (**g**) distal portion of the median duct; (**h**) setup for guano collection: energy input experiment; holding facility; (**i**) captivity; (**j**) Chamber IV (final portion)

The study was conducted in Toca do Morrinho Cave, situated at an altitude of 600 m in the municipality of Campo Formoso, Bahia, Brazil (Fig. 1), during the summer months of January and February 2013. The surrounding vegetation is dominated by deciduous species characteristic of the Caatinga biome (Ferreira et al. 2007). The regional climate is classified as hot semi-arid tropical (Köppen–Geiger BSh), with mean annual temperatures of approximately 27 °C and precipitation around 500 mm, marked by short rainy periods and prolonged droughts (Alvares et al. 2013).

The cave, developed in dolomites of the Una Group, has a single entrance leading to a horizontal projection of 475 m (Fig. 1). Its internal structure comprises three large chambers with high ceilings and a low-ceiling passage (Chamber II). *Artibeus planirostris* have occupied the first and third chambers as roosting sites for nearly three decades (Rodrigo L. Ferreira, pers. comm. 2025).

## Captivity

To quantify daily guano production per bat, a 16-day captive experiment was conducted inside the cave from 18-Jan to 2-Feb-2013, yielding 15 complete 24-hour guano samples from *Artibeus planirostris* under conditions of *ad libitum* food availability.

**Fig. 2:**
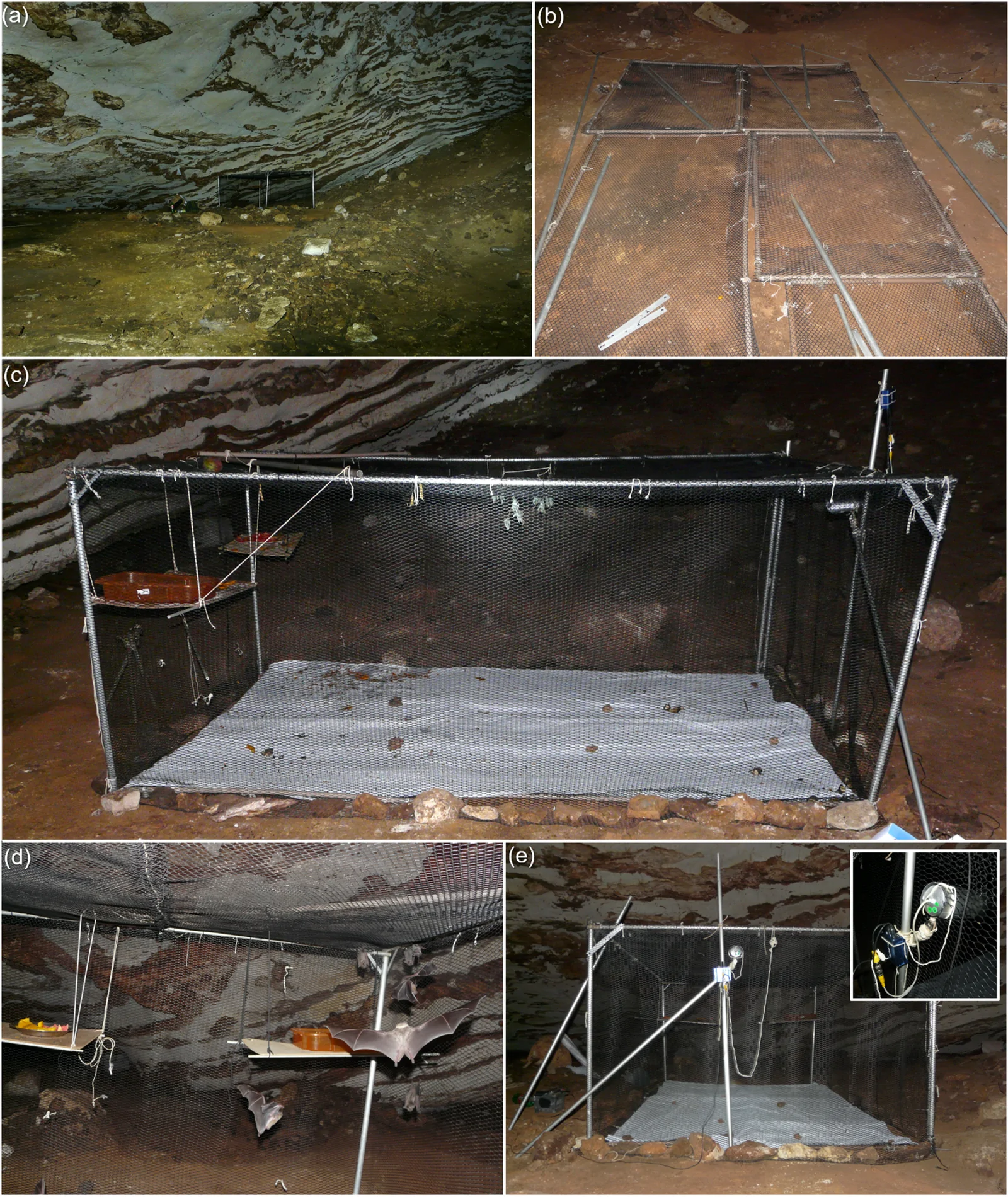
Experimental enclosure established within Toca do Morrinho Cave: (**a**) Captivity experimental; (**b**) assembly of the structure measuring 3 m in length, 2 m in width, and 1.6 m in height; (**c**) enclosure during the nighttime, with collection paper containing guano and food remains; (**d**) captive *Artibeus planirostris* individuals flying inside the enclosure, with water and food containers visible in the background; (**e**) video documentation of *A. planirostris* under captive conditions

Prior to establishing the enclosure, potential sites were assessed for microclimatic suitability. Five thermohygrometers recorded temperature and relative humidity (RH) at 30-minute intervals: four inside the cave (Fig. 1c–e, i) and one in an abandoned house outside. Monitoring was conducted for 13 days (5–17 January 2013). Mean temperatures were relatively stable across sites: outside the cave 23.13 °C (± 0.56), Chamber I 23.06 °C (± 0.59), Chamber II 21.07 °C (± 0.89), Chamber III 26.9 °C (± 0.04), and Chamber IV 26.7 °C (± 0.04). In contrast, RH showed greater variation: outside 60.68% (± 12.93), Chamber I 61.1% (± 11.78), Chamber II 79.46% (± 13.69), Chamber III 84.5% (± 0.96), and Chamber IV 99.1% (± 1.03). Although Chamber IV was spatially closest to the focal colony in Chamber III, its higher RH and deviation from natural conditions led to the installation of the enclosure in Chamber III, positioned as far as possible from the existing colony. The enclosure was constructed from 1.5-inch aluminum tubes and plastic mesh, measuring 3.0 × 2.0 × 1.6 m (length × width × height; Fig. 2a, b), allowing sufficient space for natural flight activity, considered essential to replicate typical bat behavior.

Bats were captured inside the cave using mist nets and harp traps during two capture events. The first, on 18-Jan-2013, yielded 14 adult individuals (12 males, 2 females), which were placed in the enclosure. Following the escape of two bats on the fourth day, a second capture on 25-Jan-2013 added four adults, resulting in 16 individuals (14 males, 2 females) at the end of the experiment. Only adults were included, and the low representation of females is due to the exclusion of pregnant (*n* = 5) and lactating (*n* = 15) individuals captured during the study period.

During the study period, the region was experiencing the most severe drought in the past five decades (Santos et al. 2024), which limited the availability of wild fruits for feeding bats in captivity. Only *Ficus gomelleira* Kunth & C.D. Bouché (fig) and *Prosopis juliflora* (Sw.) DC. (mesquite) were found in the field. Consequently, the captive diet consisted primarily of fruits obtained from local markets and household gardens, including *Mangifera indica* L. (mango), *Psidium guajava* L. (guava), *Musa* sp. (banana), *Solanum lycopersicum* Lam. (tomato), and *Malus* sp. (apple). Food was supplied at a minimum of 50 g per individual per day in containers positioned 1.2 m above ground level (Fig. 2b), during the bats’ peak activity period (18:00–06:00 h). Potable water was provided *ad libitum* throughout the experiment.

Guano was collected daily inside the enclosure using supports lined with disposable natural-fiber paper sheets (Fig. 2a), which retained deposits over each 24-h period. Samples were weighed while still moist inside the cave using Pesola^®^ spring scales (50 g, precision ± 0.5 g; or 100 g, precision ± 1 g), and subsequently dehydrated in a laboratory oven at 60 °C for 48 h. The total daily guano mass was divided by the number of bats present to estimate mean daily guano production per individual.

Bat acclimation to captivity was assessed through body mass measurements every five days and by continuous infrared video monitoring. Behavioral observations were analyzed using all-occurrence sampling (Del-Claro 2002), focusing on routine activities such as flight, feeding, and social interactions.

## Energy imput

**Fig. 3:**
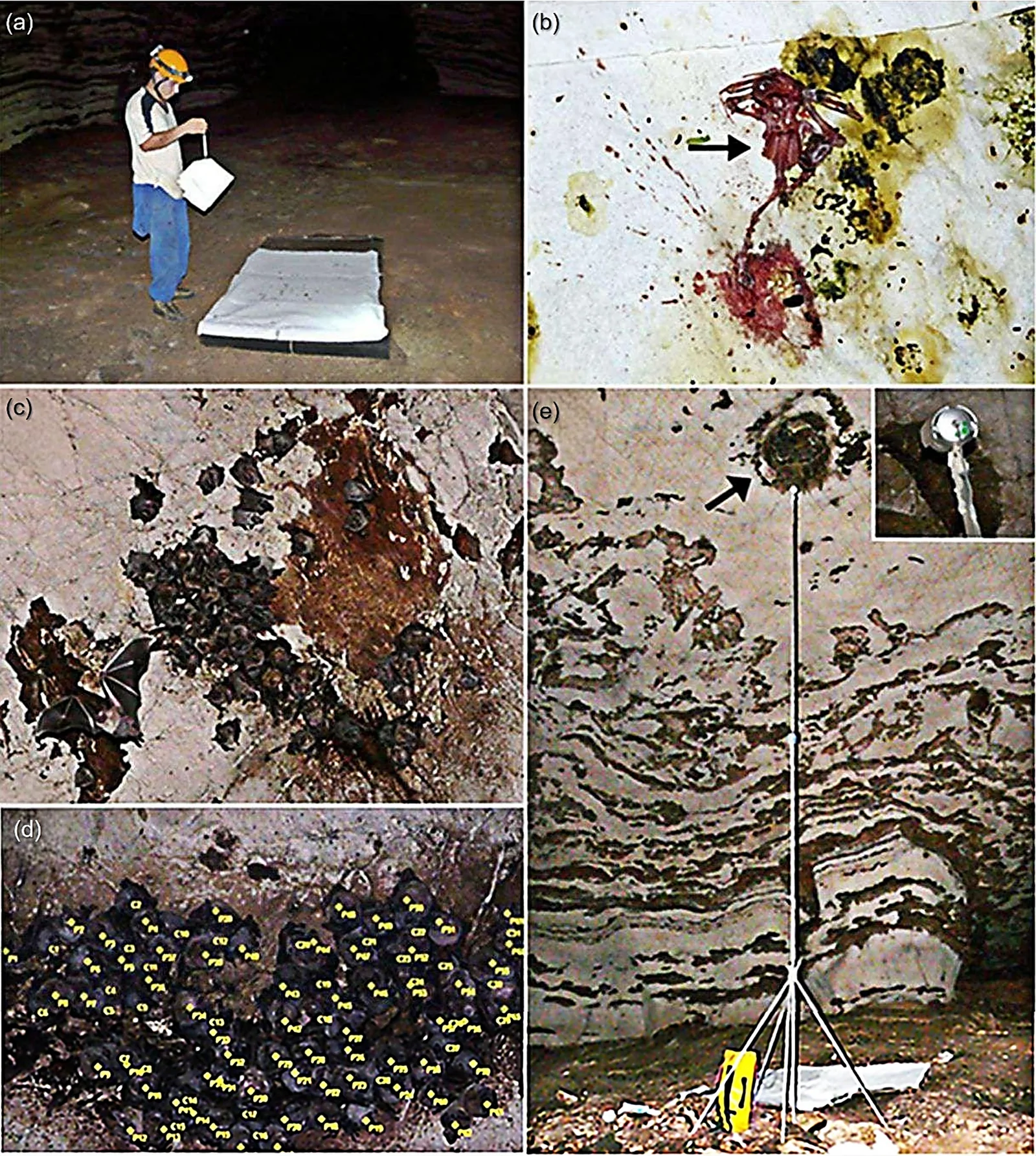
Investigation of energy input in Toca do Morrinho Cave: (**a**) support structure for guano collection; (**b**) carcass of *Artibeus planirostris* (arrow); (**c**) focal bat colony under study; (**d**) bat counting performed with Image-Pro Plus software; (**e**) video recording of the *A. planirostris* colony (arrow), with the recording device shown in detail

To quantify the energetic contribution of guano to Toca do Morrinho Cave, the average daily guano mass per *Artibeus planirostris* individual was measured over 29 days (5-Jan to 2-Feb-2013), yielding 28 complete 24-h samples (Table 2). Guano was collected using PVC pipe supports lined with disposable natural-fiber paper sheets (Fig. 3a, b) placed beneath the focal colony in Chamber III, the largest chamber of the cave. When bats temporarily split into two or three subgroups, guano was collected separately but weights and bat numbers were combined into a single daily sample.

**Fig. 4:**
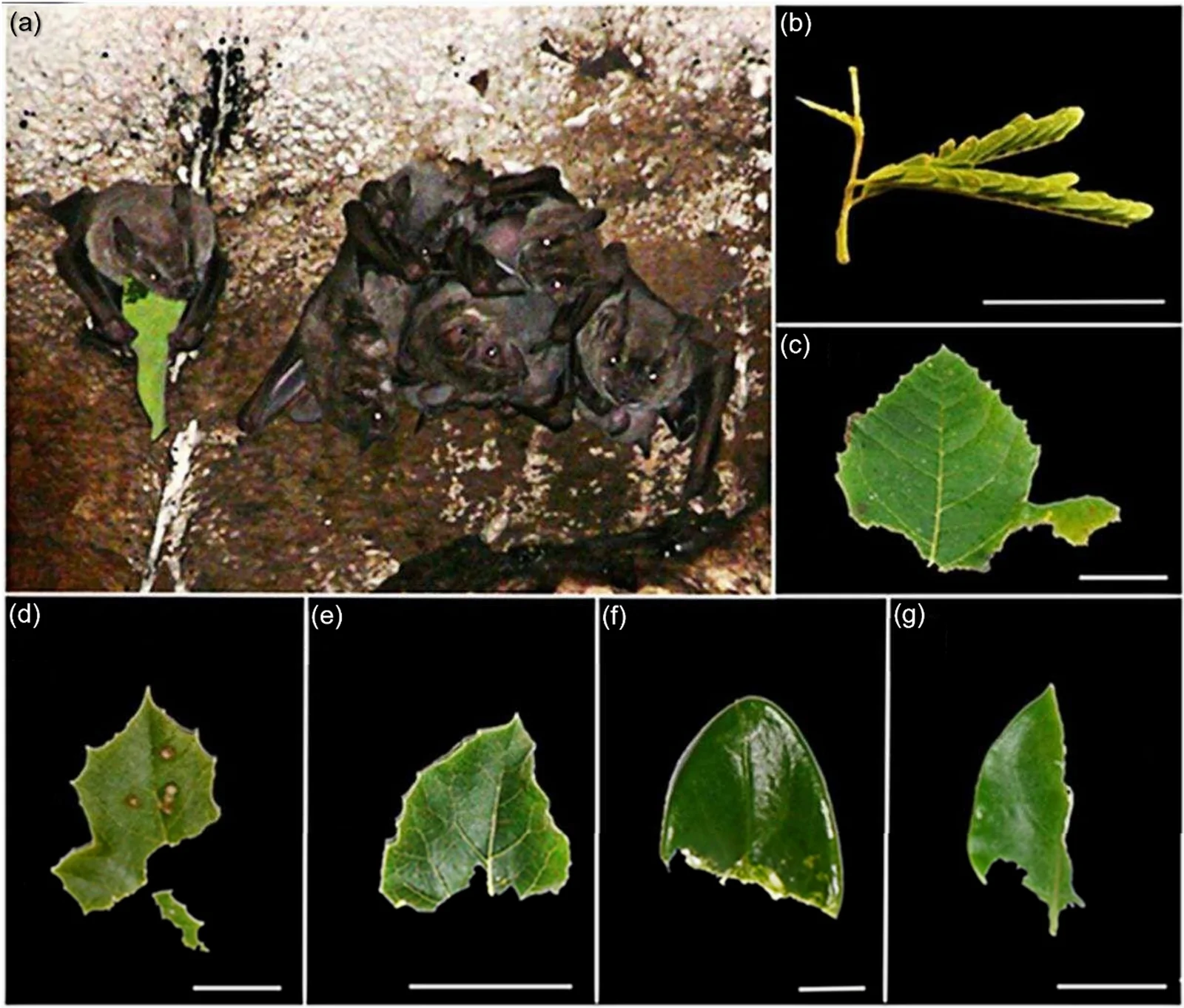
Fruit consumption by *Artibeus planirostris*, showing seeds collected from guano deposited in Toca do Morrinho Cave: (**a, b**) *Solanum* sp.; (**c, d**) *Ficus gomelleira*; (**e, f**) *Ficus* sp.; (**g, h**) Lauraceae sp.; (**i, j, k**) *Aechmea* sp.; (**l**) *Prosopis juliflora*. Scale bars: (**a, g**) = 5 mm; (**b, j, l**) = 1 cm; (**c, e**) = 1 mm; (**d, f**) = 4.5 cm; (**h**) = 2 cm; (**i**) = 3 mm; (**k**) = 5 cm

Collections were made daily at ~ 06:00 h. Guano was weighed in the field, labeled, and stored in paper bags. Seeds were separated with forceps, stored on tracing paper, and later sorted in the laboratory by morphological traits, counted, and weighed. Guano samples were oven-dried at 60 °C for 48 h to determine dry mass, with seed mass added to the final weight. Measurements were taken using Pesola^®^ spring scales (50 g, precision ± 0.5 g; 100 g, precision ± 1 g) for wet and dry guano, and an analytical balance (precision 0.0001 g) for seeds. The number of contributing adults was determined from photographic records (Fig. 3a, c) analyzed in Image-Pro Plus software (Fig. 3d). Mean daily guano production per bat was calculated as total guano mass divided by the number of contributing individuals. In addition, the presence of carcasses (Fig. 3b) and leaves carried into the cave (Fig. 4) was recorded.

Activity patterns were monitored with an infrared CCD camera (Fig. 3e) recording ~ 12 h per night (18:00–06:00 h) for two subgroups. Bats leaving the camera field were assumed to have temporarily exited the cave. Recordings were analyzed using instantaneous scan sampling (Del-Claro 2002), with 5 min sampled every 30 min and aggregated into 1-h intervals, resulting in 12 h of nightly activity data. In total, 40 h of footage were obtained, including 24 h of nocturnal activity. Recordings were made for Group I on 6-Jan-2013, and for Group II on 15 January (18:00–00:00 h) and 17 January (00:00–06:00 h).

## Seed dispersal estimates

Seed dispersal was inferred by subtracting the guano deposited inside the cave (measured from the colony) from the daily guano production estimated in the captive experiment. Seed quantities were estimated from the average number of seeds per guano sample obtained in the cave (see “Energy Input”), allowing comparisons between captive and dispersal scenarios. To account for potential changes in bat body condition under captivity (weight gain due to food surplus/reduced flight or weight loss due to stress), a correction index was applied. This index was based on the percentage change in relative body mass (forearm length/body mass) before and after captivity and used to adjust guano production per individual.

Seed viability following gut passage was evaluated through germination tests using seeds from four sources: (i) guano collected in the cave; (ii) fruits provided in captivity (control); (iii) seeds and pulp from captive bat guano; and (iv) feces deposited on leaves near the cave. Seeds represented five taxa: *Ficus gomelleira*, *Ficus* sp., *Solanum* sp., Fabaceae sp., and *Aechmea* sp. Sample sizes varied by source.

For *F. gomelleira*, germination was tested across five treatments: (i) guano from cave bats, (ii) feces from leaves near the cave, (iii) guano from captive bats, (iv) pulp from captive bats, and (v) seeds from fruits supplied in captivity (control). Each treatment consisted of four replicates of 40 seeds, totaling 800 seeds.

For *Solanum* sp., the abundance of seeds in cave guano (60,678 seeds across 29 days) allowed testing of five treatments corresponding to different collection dates (5, 10, 15, 20, and 30 January 2014). Each treatment comprised four replicates of 40 seeds, also totaling 800 seeds. Seeds of Fabaceae sp. (*n* = 15), *Ficus* sp. (*n* = 17), and *Aechmea* sp. (*n* = 26) were sown for taxonomic confirmation but were insufficient for full germination analyses.

Seeds were placed in transparent gerbox boxes (25 × 25 × 2 cm) lined with sterilized filter paper, moistened daily with distilled water. Germination was conducted under continuous light in a germinator at 28 ± 2 °C. Germination was monitored daily for 60 days, with radicle emergence ≥ 2 mm considered successful germination (Lima-Borges and Rena 1993), and seedlings followed until shoot emergence. Germination percentage and Germination Speed Index (GSI) were calculated with standard deviations. The Germination Speed Index (GSI) was calculated according to Maguire (1962), using the formula: GSI = (G₁/N₁) + (G₂/N₂) + … + (Gₙ/Nₙ) where Gₙ is the number of seeds germinated on day n and Nₙ is the number of days since sowing. This index reflects the speed and uniformity of germination, with higher values indicating faster germination (Maguire 1962). Seedlings were later transplanted to soil containers for growth and taxonomic identification.

For *Solanum* sp. and *F. gomelleira*, the experiment followed a completely randomized design. Data were analyzed using ANOVA, with percentage data arcsine-transformed [√(x/100)], and means compared using the Scott–Knott test at 5% significance, implemented in Sisvar software (Ferreira 2011).

## Results

### Captivity

Guano production by captive bats was variable (Table 1). On average, 14.7 ± 1.64 individuals were monitored, producing a total of 1,552 g of wet guano, equivalent to 752.9 g of dry matter. Mean daily guano deposition per bat was 7.16 ± 3.15 g (wet) and 3.41 ± 1.93 g (dry). The mean difference between wet and dry guano (53.7 ± 16.08%) reflected both water content and urine absorbed by the collection sheets.

**Table 1:**
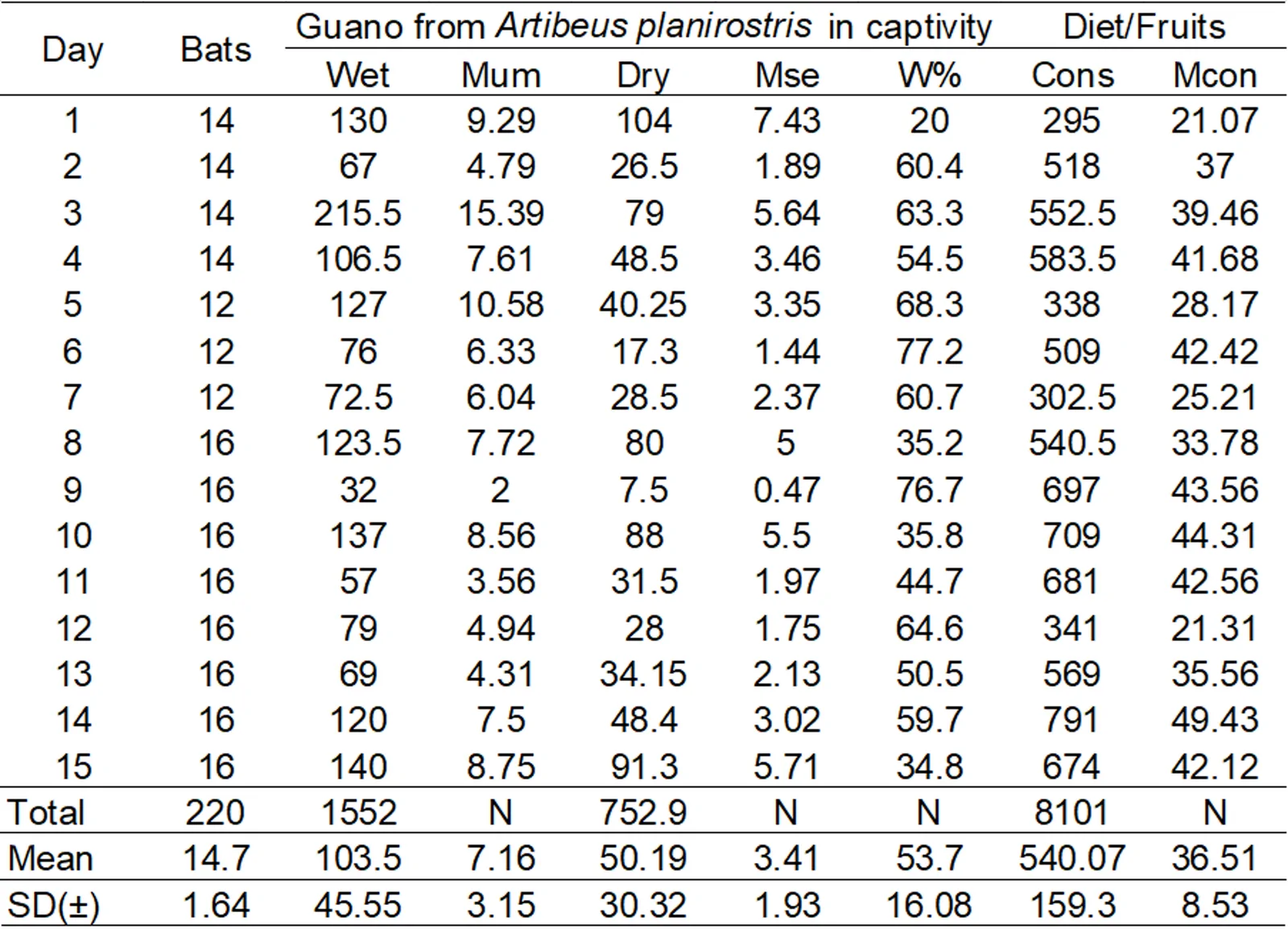
Diet and guano analysis of *Artibeus planirostris* in a captivity experiment. The table presents the number of bats monitored (Bats), the total wet guano weight (g) collected in situ and dry guano weight (g) after oven-drying, followed by the mean guano weight per bat for wet (Wet Mean, Mum) and dry (Dry Mean, Mse) samples the percentage difference between wet and dry weight (W%), and the total weight of fruits consumed (g) (Consumed, Cons) with the mean per bat (Consumed Mean, Mcon)

During the experiment, bats consumed 8,286 g of fruit, consisting of papaya (2,835 g), banana (2,694 g), mango (1,960 g), Fig. (511 g), guava (139 g), tomato (119 g), and *Prosopis juliflora* (28 g). This corresponded to an average intake of 36.51 ± 8.53 g/bat/day, approximating the mean body mass of the species (36.41 ± 5.23 g).

Bats adapted rapidly to captivity, showing a mean relative weight gain of 0.08 ± 0.2 g. Application of the correction index indicated an approximate 5% increase in guano deposition per bat. Video recordings confirmed that the enclosure permitted natural flight and foraging behavior, including the transport and handling of food items.

## Energy input

The bat assemblage in Toca do Morrinho comprised nine species from three families: Phyllostomidae (*Artibeus planirostris*,* Carollia perspicillata*,* Desmodus rotundus*,* Diphylla ecaudata*,* Lonchophylla mordax*,* Micronycteris megalotis*, and *Phyllostomus hastatus*), Furipteridae (*Furipterus horrens*), and Emballonuridae (*Peropteryx macrotis*). Functional guilds included three herbivores, three insectivores, two hematophages, and one omnivore. All species contributed to the cave’s organic input, but quantitative estimates were obtained only for *A. planirostris*.

Guano production by the cave colony was more variable than under captivity (Table 1). The number of contributing bats averaged 78.96 ± 22.2. Over the study period, they produced 1,181.5 g of fresh guano (612.48 g dry). Per capita deposition averaged 0.56 ± 0.27 g (fresh) and 0.29 ± 0.18 g (dry) per day. The difference between fresh and dry guano (54% ± 15.4) reflected moisture and urine content.

**Fig. 5:**
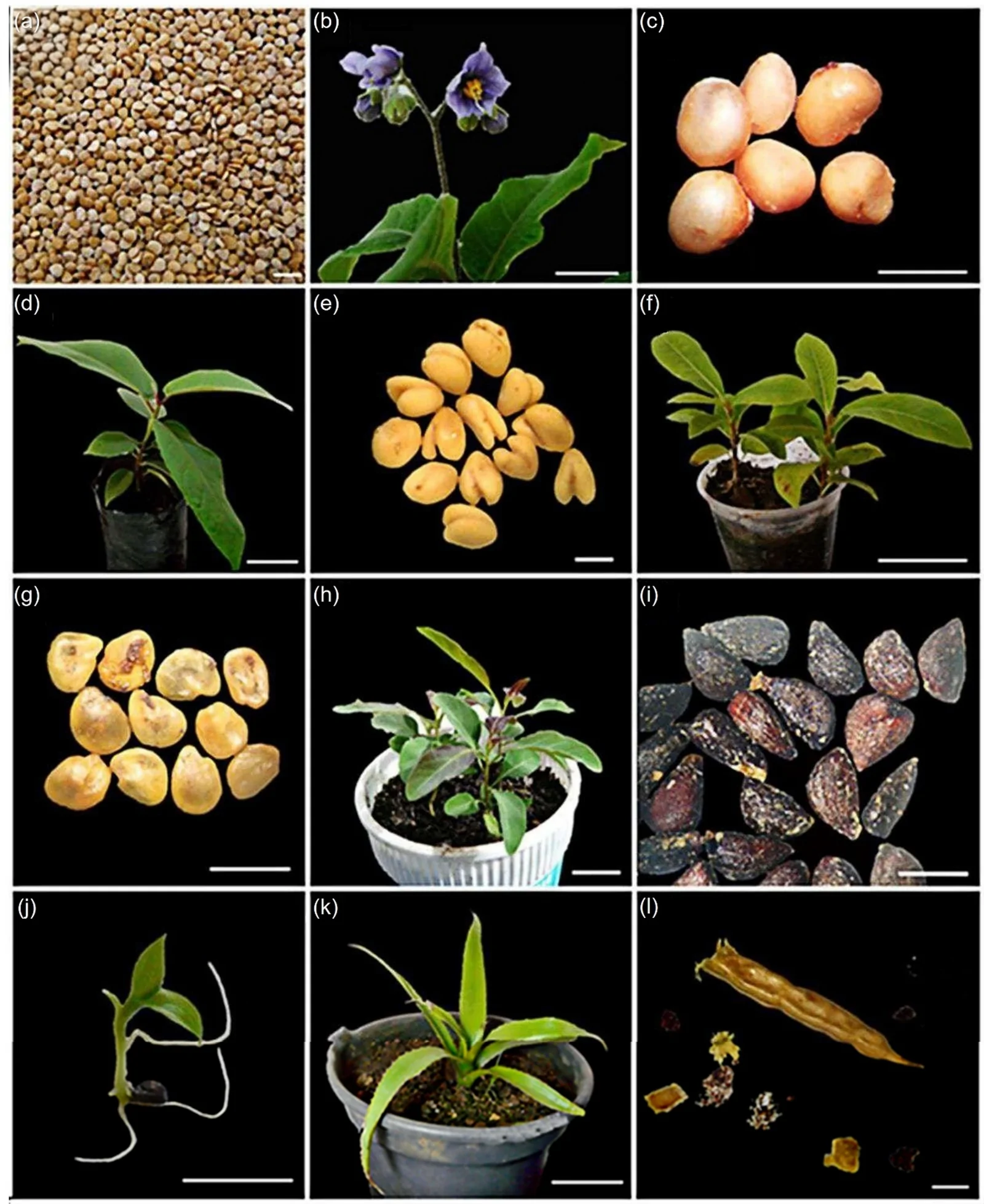
Leaf consumption by *Artibeus planirostris* in Toca do Morrinho Cave. (**a**) Focal colony; (**b**) *Prosopis juliflora*; (**c–e**) Euphorbiaceae; (**f–g**) Moraceae. Scale bars (mm): (b) 2.15; (c) 2; (d) 2.5; (e) 3; (f) 2; (g) 4.75

A total of 61,105 seeds were recovered from guano samples, with *Solanum* sp. overwhelmingly dominant (99.3%, *n* = 60,678). Only 0.18% of these seeds were partially consumed, while all others were intact. Seeds represented ~ 10% of guano dry mass (61.1 g). On average, each bat deposited 27 ± 31.56 seeds per day (Table 2). Additional organic inputs included six carcasses (four juveniles, two young) that were consumed within 24 h by *Endecous bahiensis* crickets (Fig. 3b), as well as partially eaten leaves transported into the cave (Fig. 5).

**Table 2:**
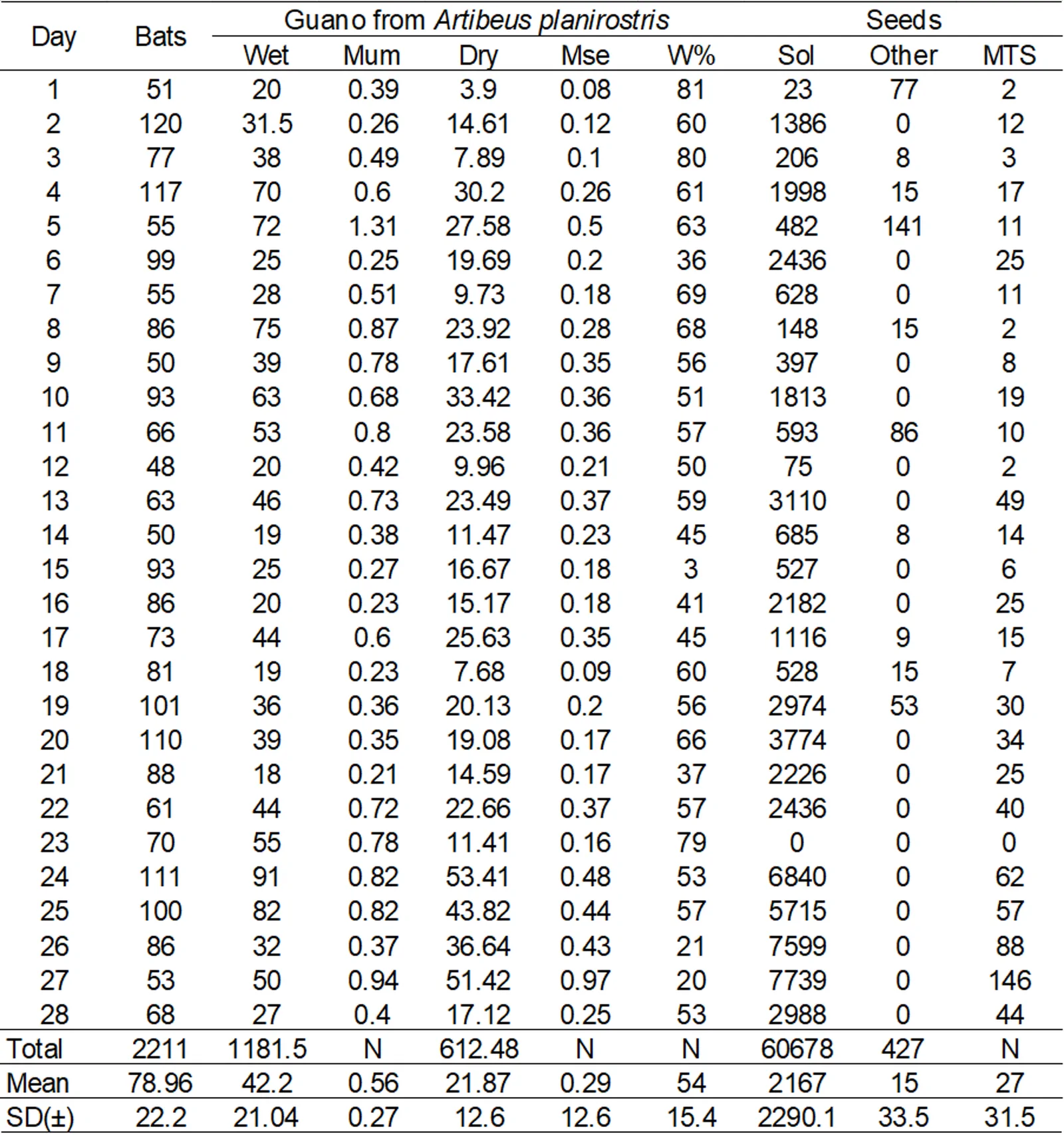
Guano deposited by *Artibeus planirostris* inside Toca do Morrinho Cave. The table presents the number of bats contributing to guano deposition (Bats), the weight of wet guano collected in situ and dry guano after oven-drying, followed by the mean wet (Wet Mean, Mum) and dry (Dry Mean, Mse) guano per bat, the percentage difference between wet and dry weight (W%), the number of seeds from *Solanum* sp. (Sol) and other species (Other) present in the guano, and the mean number of seeds per bat (Mean Seeds per Bat, MTS)

The diet of *A. planirostris* was strictly herbivorous, comprising 11 plant species. Leaves from three Euphorbiaceae and two Moraceae were identified among transported material (Fig. 5c–g). Seeds from guano indicated consumption of *Solanum* sp. (Fig. 4a, b), *Ficus gomelleira* (Fig. 4c, d), *Ficus* sp. (Fig. 4e, f), Lauraceae sp. (Fig. 4g, h), and *Aechmea* sp. (Fig. 4i–k). *Prosopis juliflora* was consumed as both leaves (Fig. 5b) and fruits (Fig. 4l), though seeds were not ingested.

**Fig. 6:**
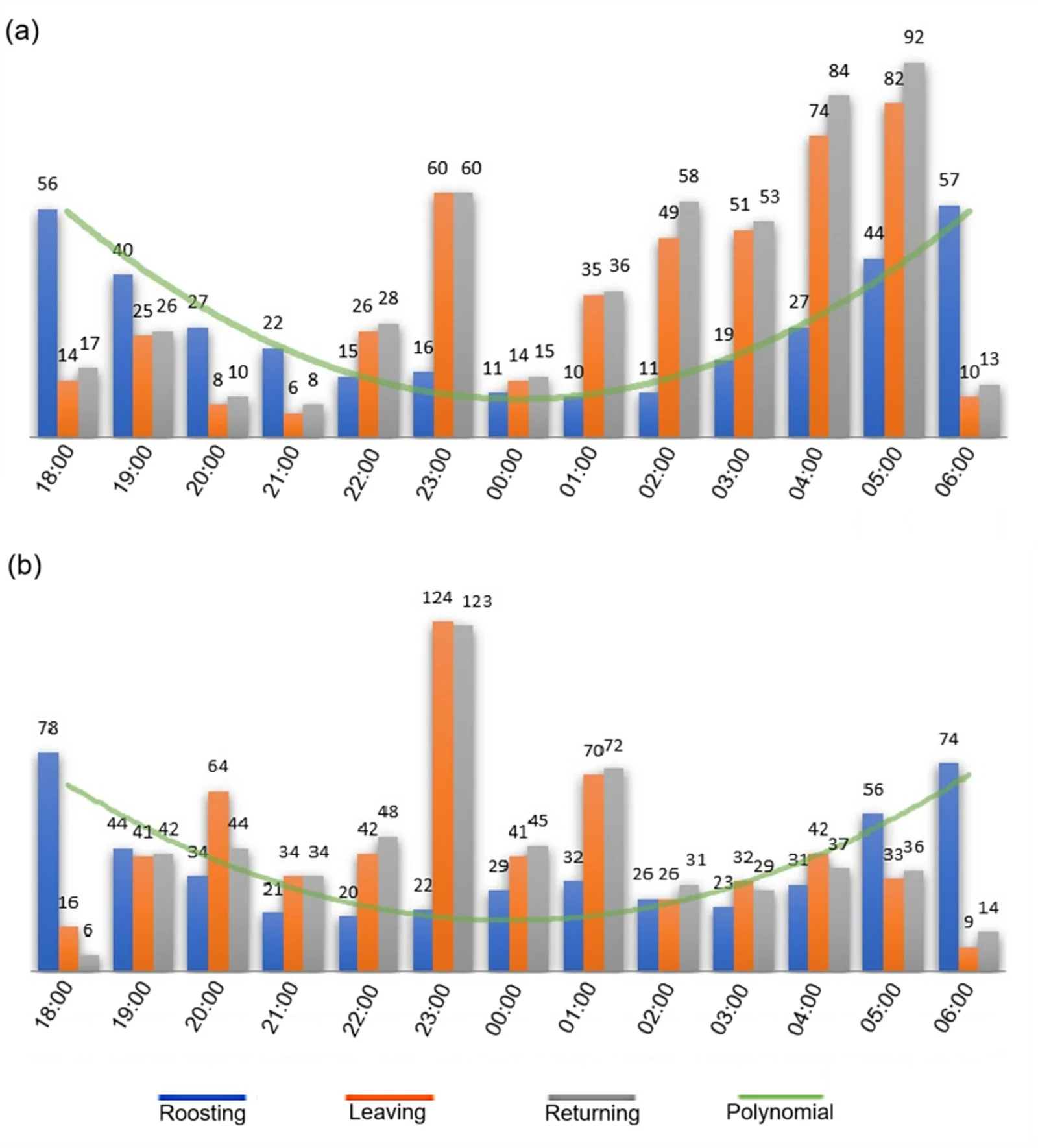
Nocturnal activity and roosting patterns of *Artibeus planirostris* in Toca do Morrinho Cave. The plots show the number of bats roosting (blue), exiting (red), and landing (gray), with the trend line for roosting bats in green. Both groups I (**a**) and II (**b**) were located in Hall III and filmed in January 2013

Nocturnal activity patterns were similar between the two groups monitored (Fig. 6). Approximately 80% of bats remained outside the cave for at least four hours (22:00–02:00), and 50% for up to nine hours (19:30–04:30). The ~ 20% that remained inside were primarily juveniles, young bats practicing flight, and lactating females returning intermittently to nurse. Occasionally, only juveniles and young individuals were present in the roost.

Females returned to nurse pups throughout the night, with three activity peaks: 18:00–20:00, 23:00–00:00, and 04:00–06:00, the latter coinciding with general colony return. Pups remained stationary, moving only when approached by mothers. Young bats displayed two peak activity periods (21:00–23:00, 01:00–02:00), during which they exercised wings and made short flights. Although capable of aerial maneuvering, many struggled to return to the ceiling, sometimes requiring > 20 attempts. Bats preferred rough surfaces with chert outcrops for landing; even adults found it difficult to perch directly at the colony site when it was densely occupied.

## Seed dispersal

From the average dry guano production per bat (3.41 g), a 5% reduction was applied using the correction index to account for increased deposition in captivity, resulting in an adjusted value of 3.24 g per bat. Based on this correction, each individual of *Artibeus planirostris* is estimated to deposit 2.95 g of dry guano daily outside Toca do Morrinho, corresponding to ~ 274.5 seeds. Thus, *A. planirostris* contributes approximately 91.05% of its guano to seed dispersal and 8.95% to nutrient input within the cave (Table 3).

**Table 3:**
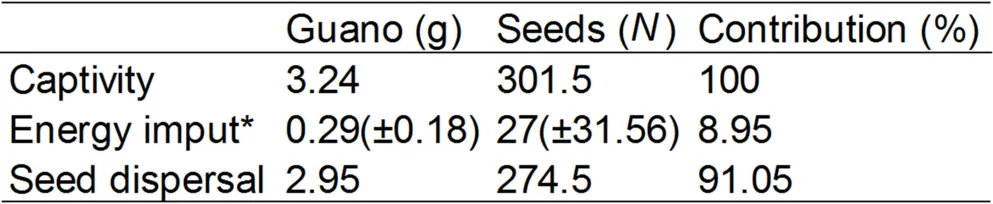
Estimated dry guano production (g) per individual of *Artibeus planirostris* and average number of seeds in the experiments: Captivity – representing total guano (corrected using a 5% adjustment index); Energy Input – representing guano deposited inside Toca do Morrinho Cave (Campo Formoso, BA); and Seed Dispersal – representing the difference between the captivity and energy input experiments. Percent contribution to each ecosystem service is also presented

**Fig. 7:**
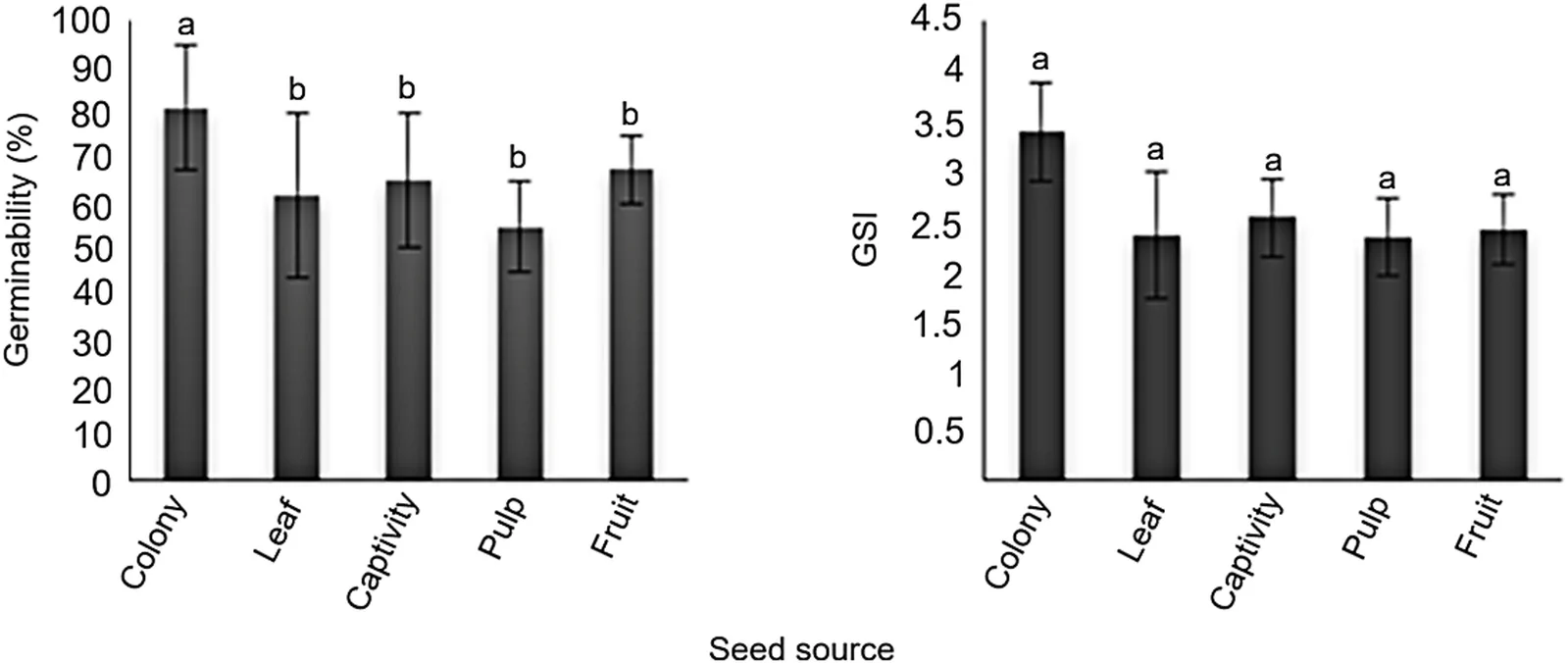
Germinability (%) (left) and Germination Speed Index (GSI) (right) of *Ficus gomelleira* seeds utilized by *Artibeus planirostris*, distributed across five treatments: bat guano from the colony (energy input); guano found on leaves; guano from captive bats; fruit pulp discarded by bats during feeding in captivity; and seeds obtained directly from mature fruits

Seed passage through the digestive tract did not reduce germination capacity. For *Ficus gomelleira*, germination of seeds from colony guano (81 ± 13.4%) was significantly higher than in all other treatments, including the control (non-ingested seeds) (Fig. 7). No significant differences were found between seeds from captive bat guano and those obtained directly from control fruits, as both originated from the same dietary source. Although the Germination Speed Index (GSI) did not differ significantly among treatments (*p* > 0.05), seeds from colony guano showed the highest GSI (3.41 ± 0.48 days) (Fig. 7).

**Fig. 8:**
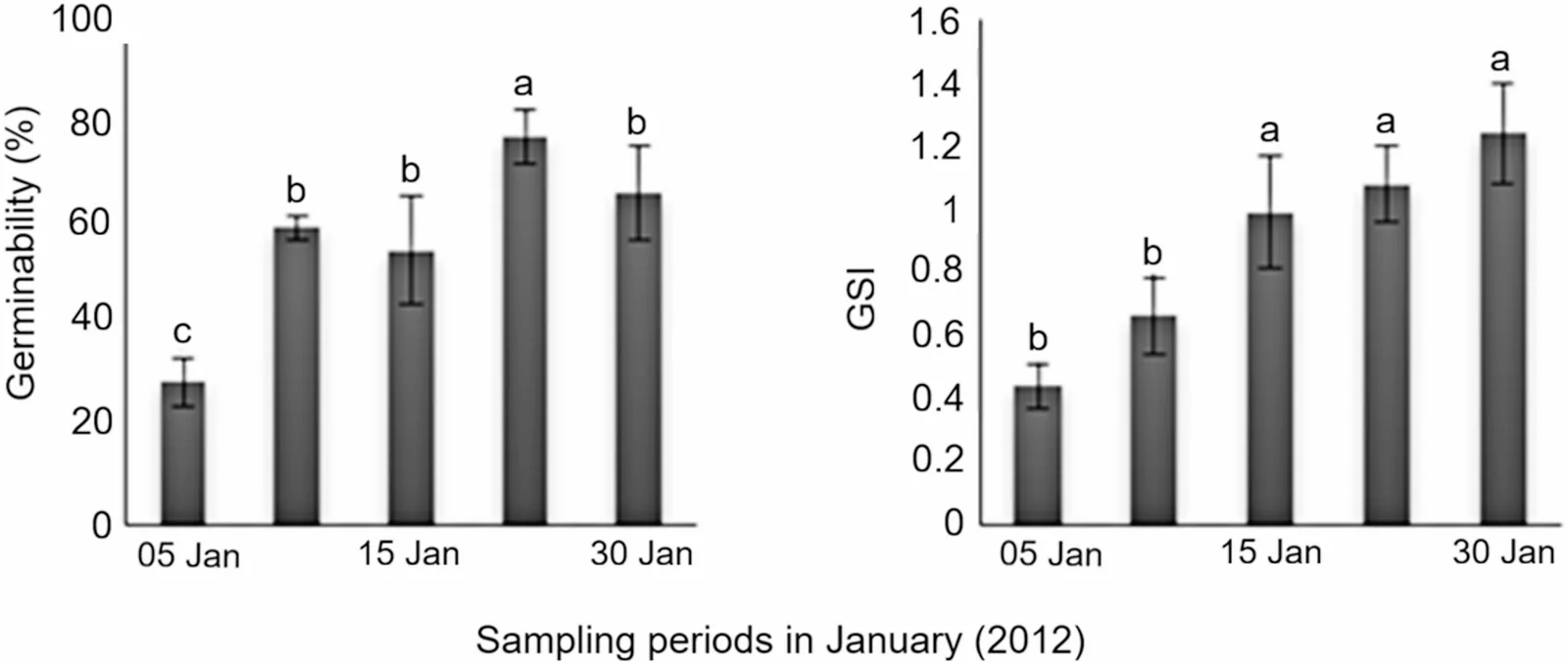
Germinability (%) and Germination Speed Index (GSI) of *Solanum* sp. (Solanaceae) seeds collected from *Artibeus planirostris* feces and stored across five time periods

For *Solanum* sp., germination success varied with collection date. Seeds collected on 5 January 2012 had the lowest germination (28.12% ± 4.74) and GSI (0.44 ± 0.07), whereas those collected on 25 January 2012 exhibited the highest values across all sampling periods (Fig. 8).

Despite limited sample sizes, other species showed moderate to high germination. *Aechmea* sp. (*n* = 26) reached 100% germination within three days and displayed the highest GSI (8.67). *Ficus* sp. (*n* = 17) achieved 88.33% germination with a GSI of 0.86, while Lauraceae sp. (*n* = 15) exhibited lower germination (33.33%) with a GSI of 0.18.

## Discussion

Organisms capable of transferring energy, nutrients, or propagules across ecosystem boundaries are increasingly recognized as cross-ecosystem engineers, as they mediate ecological processes that operate simultaneously in distinct environments (Jones et al. 1997). In frugivorous bats that roost in caves, this ecological function arises from the continuous exchange of organic matter between epigean and subterranean systems (Piló et al. 2023). While foraging in surrounding landscapes, these bats disperse viable seeds that contribute to plant recruitment and influence vegetation dynamics (Muscarella and Fleming 2007; Kunz et al. 2011). Conversely, when returning to their cave roosts, they transport substantial quantities of organic matter (primarily in the form of guano, and partially digested plant material) into subterranean habitats, thereby sustaining food webs that would otherwise be strongly limited by resource scarcity (Ferreira and Martins 1999).

Within this framework, our results indicate that *Artibeus planirostris* performs a dual ecological role, functioning both as an effective seed disperser in surface environments and as an important vector of organic matter into subterranean ecosystems. Although less than 10% of the guano produced by this species is deposited inside caves, this fraction represents a significant energy subsidy for subterranean communities. In contrast, more than 90% of guano is released outside the cave, dispersing large numbers of viable seeds with moderate to high germination potential. This pattern reflects a functional trade-off between seed dispersal in epigean habitats and nutrient transfer to subterranean systems, reinforcing the role of *A. planirostris* as a cross-ecosystem engineer linking surface and underground environments (Piló et al. 2023).

Although records of *Artibeus planirostris* in cave habitats are scarce, the species has successfully occupied Toca do Morrinho for nearly three decades, with evidence of colony presence since at least the early 1990s (Rodrigo Lopes Ferreira, pers. comm.; Ferreira and Martins 1998). The cave’s configuration offers effective protection against weather and predators, providing a secure site for roosting and reproduction not only for *A. planirostris* but also for eight additional bat species: *Carollia perspicillata*,* Desmodus rotundus*,* Diphylla ecaudata*,* Lonchophylla mordax*,* Micronycteris megalotis*,* Phyllostomus hastatus*,* Furipterus horrens*, and *Peropteryx macrotis.*

Cave-dwelling bats, such as those of Toca do Morrinho, deliver ecosystem services consistent with their trophic guilds, including pollination, seed dispersal, and the regulation of insect and vertebrate populations. Additionally, they represent the primary agents of organic matter input into this subterranean ecosystem (Ferreira and Martins 1999; Ferreira et al. 2007; Sakoui et al. 2020).

### Captivity

Captive experiments with bats are often challenging due to their sensitivity to stress and the limited capacity of many species to adapt to confinement (Bernard 1995; Esbérard and Gomes 2001). However, frugivorous bats such as *Artibeus planirostris* tend to adjust more readily to captive conditions when adequate food resources are provided (Bocchese et al. 2007; Pereira et al. 2010). In the present study, the use of a large enclosure installed within the cave likely reduced stress by maintaining natural temperature and humidity conditions, thereby allowing individuals to express behaviors typical of free-ranging bats, such as fruit transport and feeding. This semi-natural experimental setup enabled the reliable quantification of guano deposition and the evaluation of seed viability while minimizing potential behavioral disturbance.

Nevertheless, comparisons between captive and free-ranging individuals should be interpreted cautiously, as experimental conditions cannot fully reproduce the complexity of natural ecological contexts. For instance, the average dry guano mass produced by *A. planirostris* in captivity (3.41 g) was similar to values reported for *A. jamaicensis* (3.34 g; Morrison 1980). Despite this similarity, applying a correction factor to the raw experimental data remains important to account for potential biases associated with captivity, thereby improving the reliability of estimates of guano deposition under natural colony conditions.

### Energy imput

Toca do Morrinho is a permanently dry cave in which bat guano constitutes the primary resource sustaining a highly diverse invertebrate community, comprising at least 85 species across 26 families (Ferreira and Martins 1999; Ferreira et al. 2007). Analysis of the organic matter deposited by bats in this cave provided insights into the diet of *Artibeus planirostris* within the Caatinga biome, including the consumption of leaves from six plant species, a behavior previously reported for this species (Cordero-Schmidt et al. 2016). Folivory has been documented in two genera: *Artibeus*, with five species (*A. amplus*,* A. concolor*,* A. fimbriatus*,* A. jamaicensis*, and *A. lituratus*), and *Platyrrhinus*, with a single species (*P. lineatus*) (Nogueira and Peracchi 2008; Ruiz-Ramoni et al. 2011; Cordero-Schmidt et al. 2016). Unlike most bats that exhibit folivory seasonally (Kunz and Diaz 1995; Rocha et al. 2017), *A. planirostris* appears to display a non-seasonal pattern, similar to that observed in *A. amplus* (Ruiz-Ramoni et al. 2011). The underlying drivers of folivory remain uncertain, and whether leaves are consumed for nutritional or medicinal purposes has yet to be rigorously evaluated.

Moisture derived from guano and urine accounts for nearly half of the material deposited by *A. planirostris* in the cave, creating localized microhabitats. In Toca do Morrinho, invertebrate assemblages appear to depend more strongly on these moist guano deposits than on the general cave environment (Ferreira et al. 2007). Moisture also enhances the bioavailability of organic matter to invertebrates. Humphreys (1991) demonstrated that while the addition of water or organic matter alone does not restore invertebrate populations in desiccated caves, their combined presence enables recolonization and recovery of the fauna.

### Seeds dispersal

Bats are recognized as highly effective seed dispersers (Silveira et al. 2024). Unlike most frugivores, they rarely consume fruits directly at the source plant (except for very large fruits) and instead transport them to feeding roosts, often located on different plant species. This behavior, observed even under captive conditions, allows bats to visit multiple feeding sites in a single night, dispersing seeds during flight and thereby increasing the probability of deposition in favorable sites for germination (Thies and Kalko 2004; Silveira et al. 2024).

However, seed movement alone does not guarantee dispersal success, which requires seedling establishment (Schupp et al. 2010). Effectiveness depends on feeding behavior (e.g. frequency of fruit consumption, number of ingested seeds, oral processing) and the effects of gut passage (Pijl 1982). In this study, *Artibeus planirostris* consumed fruits of five plant species. Although a small fraction of *Solanum* seeds (0.18%) appeared partially chewed, likely due to fruit handling rather than seed predation, the majority of seeds were intact and viable, confirming the role of *A. planirostris* as a seed disperser rather than a predator.

Germination success of *Ficus gomelleira* seeds from captive guano and control fruits was lower than that observed for seeds from the wild colony, probably reflecting variation in fruit origin, as captive and control fruits were obtained from the same plant. Oliveira and Lemes (2010) reported that *A. planirostris* disperses *Cecropia pachystachya*, although gut passage does not enhance its germination or germination speed index (GSI). In contrast, for *F. gomelleira* they found a positive effect of gut passage on GSI. For *Solanum* sp., lower seed viability in early samples may be attributable to storage conditions or fruit origin, as prolonged storage can reduce seed vigor and germination potential, and differences in maternal plant condition may influence seed quality. Therefore, reduced viability in these samples likely reflects methodological or source-related variation rather than a negative effect of gut passage.

High seed viability appears to be characteristic of *Aechmea*. For instance, *A. bromeliifolia* and *A. castelnavii* seeds, once washed to remove mucilage, exhibited germination rates of 99% and 96%, with GSIs of 4.1 and 3.95, respectively (Silva and Scatena 2011). Although *Prosopis juliflora* seeds are not ingested, bats transport its fruits to feeding roosts, consume the pulp, and discard intact seeds. This behavior enhances dispersal by reducing fungal attack risk and minimizing density-dependent mortality near parent plants, while also enabling long-distance transport (Morrison 1978; Heer et al. 2010).

While bats consistently benefit from their interactions with plants, reciprocity is not always assured (Mello and Passos 2008). Antagonistic interactions, such as seed predation (Nogueira and Peracchi 2003; Wagner et al. 2015), folivory (Kunz and Díaz 1995; Jorge et al. 2023), or seed deposition inside caves, provide no advantage to plants and reflect more efficient exploitation of resources by bats (Morrison 1980). This study is the first to explicitly address the antagonistic effects of cave roosting on dispersal efficiency, highlighting that deposition of seeds in aphotic zones represents a significant (but relatively minor) negative outcome compared to the benefits provided by bats as dispersers.

When frugivorous bats roost in caves, a fraction of seeds is rendered non-viable due to deposition in unsuitable conditions. Nevertheless, *A. planirostris* remains an effective disperser: less than 10% of guano is deposited inside caves, while the majority (> 90%) is released in epigean habitats, underscoring its high dispersal potential. Ultimately, germination success depends not only on dispersal but also on the ecological and physiological traits of each plant species (Carvalho et al. 2017).

### Final remarks

Taken together, our findings demonstrate that cave-roosting frugivorous bats simultaneously support subterranean food webs and contribute to plant recruitment in surrounding landscapes. By dispersing viable seeds into epigean habitats while transporting organic matter and moisture into resource-limited cave systems, *Artibeus planirostris* mediates bidirectional ecological flows that link surface and subterranean environments. Through this process, the species contributes to both biodiversity maintenance and ecosystem functioning across these interconnected systems.

In semi-arid environments such as the Caatinga, where resource availability is spatially and temporally heterogeneous, this functional connectivity may play an important role in enhancing vegetation resilience while sustaining invertebrate communities that depend on guano-derived resources within caves. From a conservation perspective, protecting cave-dwelling bat colonies should therefore be understood not only as safeguarding roosting sites but also as preserving key ecological processes that support ecosystem services across coupled surface–subterranean landscapes.

## Data Availability

The data supporting this article are archived in the Dryad: http://datadryad.org/share/WwCrXwQ-H10su1EUHCPiaqcWVP5p0AKl7f4BIWfi69U.
